# Digital therapeutics and the need for regulation: how to develop products that are innovative, patient-centric and safe

**DOI:** 10.1186/s13098-022-00818-9

**Published:** 2022-04-01

**Authors:** Marcela Rassi-Cruz, Fernando Valente, Maria Vanina Caniza

**Affiliations:** 1 Endocrinologist and Medical Head of Axenya, Rua Leopoldo de Bulhões, 59, Vila Clementino,, 04022-020 São Paulo, SP Brasil; 2 Endocrinology Division, Department of Internal Medicine, Faculdade de Medicina Do ABC, Santo André, São Paulo, Brazil; 3 Global Chair of the Healthcare & Life Sciences Group, Baker & McKenzie, Cecilia Grierson 255, 6th Floor, C1107CPE Buenos Aires, Argentina

**Keywords:** Digital therapeutics, Digital health, Regulation

## Abstract

**Background:**

Digital therapeutics are defined as therapeutic interventions that are driven by high quality software programs to prevent, manage or treat a medical disorder. These products provide great potential to improve patient outcomes, particularly for chronic disease sufferers, including people with Diabetes.

**Main text:**

As yet, regulatory pathways for these products are rather unclear across all jurisdictions, although somewhat more progress has been made in the US and UK. Since digital therapeutics use cutting-edge technology and a logic of continuous innovation, regulation used for medical devices may not be completely appropriate. However, these products could present risks to patients if not developed and used appropriately. In the article, we consider the importance of a regulation framework and the role of self-regulation by developers as a way of ensuring patient safety while promoting innovation. We particularly emphasize the inclusion of doctors and other medical professionals in the design of the products, not only as a way of ensuring safe and effective applications, but also to encourage their take-up by patients, who tend to have high levels of trust for their HCPs.

**Conclusion:**

Developers of digital therapeutics have the duty to create products that are safe, ethical and effective, without waiting for government regulation. Further, by self-regulating, following principles such as those provided by the Digital Therapeutics Alliance, they can develop products that serve patients better, while continuing to innovate.

## Background

With the arrival of digital therapeutics (DTx), digital health products are becoming more and more sophisticated—closer to medicines than to apps. Digital therapeutics use technology like data analytics, artificial intelligence and an understanding of behavioral psychology to provide an alternative to current typical methods of chronic disease treatment. They can collect health data in real time, all the time, via wearable devices and applications, and communicate it, pre-analyzed, to health care practitioners and patients, providing them with an infinitely more complete understanding of the patient’s condition and habits to inform care plans. They enable patients to maintain frequent contact with health care professionals (HCPs), to keep them motivated and help them take specific actions when required. Both parties come to appointments with reliable information and can use the consultation to develop, together, a much more compliable care plan, with the HCP aware of progress and able to take actions to motivate or change the course of treatment if necessary. Their potential to improve the management of chronic diseases, such as Diabetes, is huge. However, they continue to present something of a grey area in terms of regulation. How should developers of these exciting new products maximize their ability to improve patient outcomes and continue to innovate while ensuring patient safety? In this article we consider the benefits and potential risks of digital therapeutics, the current regulatory situation, the responsibility of developers to “self-regulate”, and ways they can do this—including by setting the HCP front and center.

## Main text

After experiencing a serious diabetes complication, which left her hospitalized, Fernanda^1^ received what seemed like a helpful piece of advice: to download a mobile health application (app) to help with carbohydrate counting. Given the fact she had just had diabetic ketoacidosis, a condition triggered by low insulin levels, the 24-year-old Brazilian primary school teacher accepted. It appeared to be a simple way to ensure that she took the right amount of insulin and avoided further issues. Unfortunately, this was not the case. Just a few weeks after starting to use the app, Fernanda experienced a severe—and avoidable—hypoglycemia. The app had recommended her to take more than twice the correct amount of insulin.

Fernanda is gradually improving her health, working alongside a nutritionist, three years after that episode. But as well as the severe health risk that she suffered in the moment, the incident generated a long- term impact on Fernanda’s wellbeing and ability to live a full life. Since it took place, she has started experiencing high levels of anxiety due to her condition. Scared to suffer from another complication, Fernanda now uses just 30% of the recommended amount of insulin to manage her disease, exposing her to hyperglycemia which is associated with long-term health deterioration.

The case is shocking, but perhaps not surprising. A paper published last year in the Academic Journal of the American Medical Informatics Association reviewed recent research literature to identify 80 different safety concerns related to digital health applications [1]. These ranged from the provision of incorrect or misleading information (with advice both bizarre and horrifying, such as that to bipolar disorder sufferers to “take a hard shot of liquor before bed”), to incorrect calculations and diagnostic outputs, and, most concerning, an inappropriate response to patient needs, such as extremely high scores of shortness of breath, pain, or suicidal ideation. Over three-quarters of the safety concerns reported were associated with “potentially hazardous” outcomes. It is unclear whether Fernanda’s experience was due to a fault or an app bug, or just down to human error—another ever-present risk for products that are unprescribed and that put the collection of information in the hands of the patient themselves.

The potential risk has been around ever since digital health applications could be downloaded by anyone with a smartphone—as far back as 2011, one of the biggest pharmaceutical companies in the world was forced to withdraw its rheumatology calculator app after errors were found in its calculation formulas. Ten years later, there is an exponentially larger number and variety of applications—over 350,000 are currently available to consumers according to a recent IQVIA study [2]. In general, these are either wellness-related, or what the US Food and Drug Administration regulatory body (FDA) classifies as “low risk” applications. They are simple to download from the App or Play Store without any prescription, qualification, or oversight—and the potential for damage is likewise generally fairly limited.

However, the latest generation of digital health applications—digital therapeutics or “software as medical device” (SaMD)—are far more advanced in their ability to diagnose and offer treatment advice. According to the Digital Therapeutics Alliance (DTA), digital therapeutics “deliver evidence-based therapeutic interventions that are driven by high quality software programs to prevent, manage, or treat a medical disorder or disease. They are used independently or in concert with medications, devices, or other therapies to optimize patient care and health outcomes” [3].

### Challenges in chronic diseases treatment—the impact of DTx on data analysis

DTx are varied but tend to work with chronic diseases or neurological disorders. Chronic noncommunicable diseases have become the biggest killers in Latin America and across the world. According to the World Health Organization (WHO), they now account for seven of the ten main causes of death globally [4]. In Latin America a third of adults suffer from at least one chronic disease, while two thirds of deaths are related to one or various of them [5]. Yet, even as they become more prevalent, they are still poorly served by most healthcare systems, which are set up to manage acute illness: sudden, with clear symptoms and of limited duration, to be dealt with by one or a small number of discrete medical interventions. Chronic illness on the other hand is often characterized by a slow onset and unclear symptoms, and requires monitoring and attention for the long-term or even for life. Trying to deal with the latter with a system set up for the former means that treatment for chronic disease can be fearsomely inefficient, leading to repeating cycles of worsening health, leading to hospitalizations or emergency interventions.

That is because the current ill-care model relies on lone data points collected during a medical appointment, often at three month-intervals, or even further apart. Everything one with chronic disease experiences outside those half-hour interactions—specific symptoms, lifestyle choices—are down to them to identify, remember, record and correctly (and honestly) disclose to the physician during the consultation, and for the health care professional (HCP) to correspondingly interpret and respond to. This means important data often go missing, precise evolution of certain conditions is lost and problems are identified too late. Communication with the doctor focuses on data-gathering at the expense of discussion of options. Data to develop a care plan is sparse. Adherence to the plan—which often requires the patient to make disagreeable decisions—is completely up to them.

Digital therapeutics use technology like data analytics and artificial intelligence and an understanding of behavioral psychology to provide an alternative. They can collect health data in real time, all the time, via wearable devices and applications, and communicate it, pre-analyzed, to health care practitioners and patients, providing them with an infinitely more complete understanding of the patient’s condition and habits to inform care plans. They enable patients to maintain frequent contact with HCPs, to keep them motivated and help them to take specific action when required. Both parties come to appointments with reliable information and can use the consultation to develop a care plan together, which in turn is more likely to be adhered to, with the HCP aware of progress and able to take action to motivate or change as necessary. The potential impact on health and quality of life for chronic disease sufferers is huge.

And that’s before we get on to the potential impact at the system level. Chronic disease is a huge drain on the world’s health systems coffers, which are under even greater pressure. This is particularly the case in Latin America, which suffered from increasing demand, constrained healthcare budgets, fragmentation of the healthcare system and inefficient use of resources even before Covid-19 struck. Chronic disease accounts for US$500 billion of healthcare expenditure per year in Latin America alone [6]. Digital therapeutics help patients make wise, data-driven choices to better manage their illness, and thus help them to avoid extremely costly emergency treatment or hospitalization. Thus their potential is huge: not just to reduce the human costs of chronic disease, but also to make a dent in the enormous economic costs that it represents.

The previously mentioned IQVIA report identifies over 250 DTx or Digital Care (DC) products, with around 150 of those commercially available. But the potential flip side of their vast increased sophistication and capability is the worry of increasing patient risk. What’s more, with every case like Fernanda’s, doctors and patients alike are more wary of using digital health products, and the credibility of DTx takes a hit. Needless to say, Fernanda has sworn against using any kind of digital health product ever again. Given the huge potential that DTx have to improve individual patient outcomes, and healthcare at a system level, a lack of trust in this technology would be a tragedy.

## DTx regulation: a framework under construction

The IQVIA report identified “at least” 25 DTx products which have now been granted market authorization through regulatory processes, variously in the US, Europe and Japan, with another 23 commercially available. It noted that 20 of these are available by prescription only.

In general, regulatory and public health bodies are still defining what to do with these new tools. For instance, what are they? Should they be approached from a regulatory, legal and public health standpoint in the same way as pharmaceuticals? Medical devices? Consumer applications? On a global level, despite the efforts of the DTA and others, there isn’t even a common nomenclature (with different agencies using the expressions DTx, SaMD, Medical Device Software (MDSW) and others, usually to mean the same thing) or agreement on definitions. There is also a plethora of different potential focal points—on user safety, on security and privacy of personal data, on the validity of medical claims—while DTx’s various constituent parts can be problematic in and of themselves (both the International Coalition of Medicines Regulatory Authorities and the World Health Organization have recently published guidance on the use of Artificial Intelligence in healthcare) [7, 8].

Even those developers under the jurisdiction of the FDA, perhaps the most advanced regulatory world body, do not have a completely clear path. This agency conceives DTx as medical devices and is approaching their regulation accordingly, using risk classification to define which pathway each should use, which in the most extreme cases means a full 501(k) clearance [9]. On the one hand, the digital health applications available for access on all smart phones have generally come under little or no regulatory scrutiny. The more novel a product and the greater its potential risk, the more likely the FDA will apply the same regulatory pathway as it would to a standard medical device. For example, Pear Therapeutics’ reSET product for substance abuse, generally considered the first FDA-approved DTx, had to submit results of a randomized controlled trial (RCT) in order to receive regulatory approval [10]. These are lengthy and burdensome processes, particularly for digital health products that generate much of their value precisely through their rapid and innovative response to customer feedback. Meanwhile, those products that can’t easily be categorized as either DTx or consumer wellness applications, which meet the definition of medical devices but present lower risk, find themselves in a grey area of “enforcement discretion” [11].

In response to the specific characteristics of digital health products, the FDA is piloting a Pre-Certification program that aims to provide a new approach to regulation for SaMD, including a more rapid initial response, and ongoing monitoring and data collection to support what is likely to be a continually iterating product [12]. The agency appeared to confirm its commitment to digital therapeutics by relaxing some regulatory requirements to address particular health problems aggravated by the pandemic and its immediate consequences. For example, it temporarily expanded patient access to digital therapeutics for psychiatric disorders, and allowed limited modifications to certain remote monitoring devices used by chronic sufferers [13].

The UK’s National Health Service (NHS) launched in February 2021 its Digital Technology Assessment Criteria (DTAC), to be used by both developers of digital health tools, and by those in the NHS that will eventually be seeking them, including criteria around safety, data protection, technical assurance, interoperability and usability. They also require a CE or UKCA mark, putting them in the same category as traditional medical devices [14, 15]. Post pandemic and post Brexit, the Medicines and Healthcare Regulation Agency (MHRA) is in the process of developing a new delivery plan, although there are no clues yet as to what that may mean for digital health. Meanwhile, in the European Union, where DTx are also defined as medical devices, new legislation appears to mean that a greater number of new DTx products will have to provide clinical data to gain market access [16].

In Latin America things are still nebulous. In most jurisdictions, DTx have the potential to be regarded as medical devices and therefore should, at least theoretically, be subject to the same rules, but there are no clear standards for digital health [17].

In Mexico, there is currently no regulation for these products, and as of August 2021, Mexico’s National Health Regulator COFEPRIS stated that it had not yet issued a marketing authorization for any medical application. However, a new draft technical standard on good manufacturing practices, which incorporates a definition of SaMD, is expected to be published in the country this year [17].

In Colombia, the regulator Invima has considered some cases in which software, including apps, is considered a medical device, for example patient medical data analysis and interpretation, or software that sets alarms in biomedical equipment, although again, it has no specific regulation yet for this type of product [17].

Further south in the region’s largest market, Brazil, the regulator ANVISA has no specific pathway either, although a public consultation on digital health is currently ongoing, so there may be clearer guidelines in the near future. Brazil has been somewhat slow in the recognition of digital health. Only in 2020 was the use of telemedicine broadened from emergency only, and the online sale of prescription products authorized, both changes hastened by the Covid-19 pandemic [17].

The most likely outcome in the case of DTx in Latin America and worldwide is that sooner or later they will move to a regulatory model more fitting for therapeutic products. In a post-pandemic world, we may even witness new levels of cooperation and harmonization between regions that consider the virtual nature of these products, and design legislation to be used across jurisdictions. But, as reflected by the FDA’s attempts to create a new regulatory pathway for this type of product, DTx pose particular challenges for a “typical” regulatory approach.

## First, do no harm

Of course, regulation always lags behind innovation. In a VUCA (volatility, uncertainty, complexity, ambiguity) world, the continual appearance of cutting-edge technologies and disruptive business models means that regulation and legislation are practically always on the back foot. To quote a World Economic Forum report from 2016, “Given the Fourth Industrial Revolution’s extraordinarily fast technological and social change, relying only on government legislation and incentives to ensure the right outcomes is ill-advised. These are likely to be out-of-date or redundant by the time they are implemented” [18]. DTx are built around new technologies like data analytics, AI, VR and machine learning, and by definition follow the continuous innovation logic of consumer-focused digital products. How to regulate a product that doesn’t offer a new version every two years, but rather every two months?

In a sense, this is how it should be—innovation that was constrained from the start by possible regulation and legislation would be poor innovation indeed. Regulation is often considered to delay the development of new ideas. The state of the art enterprises proposed in their time by Airbnb, Uber and Amazon among others, may well not have come about if regulators had got there first. And whatever the possible repercussions and even dilemmas that are now arising from their business models, few can argue with the value that these firms have created, quite apart from the benefits to the average citizen.

Of course with DTx, unlike Airbnb or Uber, the risk goes far beyond an overpriced holiday rental or a rude driver, to human life itself. If clear regulation for DTx is always playing catch-up to some extent, how to ensure safe and ethical products? This is where “self-regulation” comes in. Put simply, developers of these products have the duty to find their own ways to reduce risk and maintain ethical standards: which will likely also help them to improve patient outcomes, and to continue developing trust and confidence among medical and patient communities that will eventually enable DTx to meet their full potential.

The DTA asks its members to abide by its decalogue of principles in their development of DTx, planned to promote the design and commercialization of safe, secure, ethical products that do what they say they will [19]. These principles include, of course, the duty to be “reviewed and cleared or certified by regulatory bodies as required” and to “incorporate patient privacy and security”, but also ways to build in safety and patient-centricity further upstream in the design process. For example, they suggest that developers “incorporate design, manufacture, and quality best practices”, and that they “engage end users in product development and usability processes”.

One simple principle of self-regulation is to put the HCP front and center in the development process and beyond. After all, the natural regulator of the product is the one who will be using it, particularly when that person is an expert. This may sound like a given in a software industry that talks so much about the user experience. And yet, most digital health apps are developed without medical professional involvement. A 2018 University College London study found that just 20% of the health apps they reviewed had been developed with participation of health experts [20].

## Just what the doctor ordered

Sometimes typecast as technology laggards, recent research suggests that HCPs see significant benefit in digital health products. A pre-pandemic American Medical Association (AMA) study found that 87% of physicians believe that digital health would offer some or a definite advantage to the care that they are able to provide for their patients [21]. The 2020 Ipsos Digital Doctor report estimated that, worldwide, 46% of doctors have recommended some form of digital health solution to their patients [22]. And of course, the pandemic gave everything a nudge: a mid-2021 McKinsey survey of US-based physicians discovered that 58% viewed telehealth more favorably than they had done before the pandemic, and that 57% would prefer to continue offering virtual care even after the lifting of pandemic restrictions [23].

But HCPs want to be active participants in the digital revolution, not just observers. In the AMA study, 42% of respondents said they would want to be responsible for the future implementation of digital health solutions and a further 47% at least consulted. One of the drivers of perceived resistance to technological change on the part of the medical profession appears to be the tendency by developers and project managers to prioritize whizzy technology over understanding of physician and patients needs and workflow. A survey carried out by Bain and HBR in 2017 found that doctors do not generally feel involved in key decisions around adoption of new models [24].

Making HCP involvement part of good design principles for DTx could both improve patient outcomes and encourage physician enthusiasm. And physician enthusiasm is no small thing: particularly in Latin America, the physician holds a large amount of sway in patients’ decisions. According to a 2018 Ipsos poll, 79% of people in Colombia, and 77% in Argentina, would use a connected health device if it was recommended by their physician [25].

One of the great possibilities of digital therapeutics is providing synchronous and ongoing communication between patients and HCPs. This isn’t just important for resolving issues or catching problems before they progress too far. It’s also likely to lead to better patient outcomes through higher motivation and greater adherence. We’ve known for a while that primary care relationships characterized by high levels of trust lead to improved patient outcomes. For example, research shows that patient adherence can be as much as three times higher when this kind of relationship is in place [26]. And this seems to translate into the digital sphere.

One digital care provider reports that the members who interact with their care team or community in the first week of the program are 24% more likely to achieve their health goals, those who message their care teams are twice as likely to achieve positive health outcomes and those who complete goals with care team support are 250% more likely to achieve outcomes [27]. This is certainly consistent with reciprocity theory, the idea that people will respond favorably to those who have helped them in the past by returning benefits for benefits, and retaliate against those who have been detrimental with either indifference or hostility [28]. Furthermore, investigation into the concept of reciprocity suggests that behavioral responses are influenced not only by an action, but by the intentions behind it [28]. Thus people will evaluate the kindness of its action not only by positive consequences but also by their perception of the intentions behind the action. Critically, if intentions are absent, the response will be less intense [28]. So, just as in the case above, people behave differently when they experience actions of real people, rather than just a bot or algorithm, for example when a digital therapeutic works with a real-life HCP, rather than the automatically generated content provided by a typical consumer application.

## Conclusions

Digital therapeutics are likely to become a critical player in healthcare in the twenty-first century. They could mean a turnaround in the way we approach the fight against chronic diseases—and the results we achieve. But given the massive and increasing pace of technological change and the nature of DTx products, government agencies are catching up to define the most suitable regulatory pathways. In the meantime, developers must take the initiative to ensure their products are ethical, safe and work well. By self-regulating, putting the patient at the center and involving HCPs in design and supervision of use, developers can help ensuring greater levels of safety and better outcomes for patients, higher levels of trust and therefore of confident recommendation from HCPs, and may eventually help to ensure that DTx meet their full potential.

## Data Availability

Data sharing is not applicable to this article as no datasets were generated or analyzed during the current study.

## Abbreviations

ANVISA: Brazilian Health Regulatory Agency
COFEPRIS: Federal Commission for the Protection Against Sanitary Risk (Mexican regulatory body)
DTA: Digital Therapeutics Alliance (industry body)
DTx: Digital therapeutics
FDA: US Food & Drug Administration (regulatory body)
HCP: Health care providers
MDSW: medical device software
NHS: UK National Health Service
SaMD: Software as a medical advice
VUCA: Volatility, uncertainty, complexity, ambiguity (used in leadership theory to refer to situation of constant change)
WHO: World Health Organization

## Footnotes

1 * Names have been changed to protect confidentiality.
